# Further validation of the association between *MAPT* haplotype-tagging polymorphisms and Alzheimer’s disease: neuropsychological tests, cerebrospinal fluid biomarkers, and *APOE* genotype

**DOI:** 10.3389/fnmol.2024.1456670

**Published:** 2024-09-25

**Authors:** Mirjana Babić Leko, Ena Španić Popovački, Nanet Willumsen, Matea Nikolac Perković, Nikolina Pleić, Klara Zubčić, Lea Langer Horvat, Željka Vogrinc, Marina Boban, Fran Borovečki, Tatijana Zemunik, Rohan de Silva, Goran Šimić

**Affiliations:** ^1^Department for Neuroscience, Croatian Institute for Brain Research, University of Zagreb Medical School, Zagreb, Croatia; ^2^Reta Lila Weston Institute, Department of Clinical and Movement Neuroscience, UCL Queen Square Institute of Neurology, London, United Kingdom; ^3^Division of Molecular Medicine, Ruđer Bošković Institute, Zagreb, Croatia; ^4^Department of Biology and Human Genetics, School of Medicine, University of Split, Split, Croatia; ^5^Department of Molecular Diagnostics and Genetics, Dubrava University Hospital, Zagreb, Croatia; ^6^Laboratory for Neurobiochemistry, Department of Laboratory Diagnostics, University Hospital Centre Zagreb, Zagreb, Croatia; ^7^Department of Neurology, University Hospital Centre Zagreb, Kišpatićeva, Zagreb, Croatia; ^8^School of Medicine, University of Zagreb, Zagreb, Croatia; ^9^Department for Functional Genomics, Centre for Translational and Clinical Research, University of Zagreb Medical School, Zagreb, Croatia; ^10^Department of Nuclear Medicine, University Hospital Split, Split, Croatia

**Keywords:** Alzheimer’s disease, *APOE* gene, biomarkers, cerebrospinal fluid, dementia, *MAPT* gene, *MAPT* haplotypes, *MAPT* polymorphisms

## Abstract

**Introduction:**

Genetic studies have shown that variants in the microtubule-associated protein tau (*MAPT*) gene, which encodes tau protein, can increase the risk for Alzheimer’s disease (AD). Additionally, two haplotypes of the *MAPT* gene (H1 and H2) are associated with various neurodegenerative disorders, including AD. This study aimed to test the association of *MAPT* haplotypes (H1 and H2) and *MAPT* haplotype-tagging polymorphisms (rs1467967, rs242557, rs3785883, rs2471738, del–In9, rs7521) with AD.

**Methods:**

The study included 964 individuals: 113 with AD, 53 with mild cognitive impairment (MCI), 54 with other dementias, and 744 healthy controls.

**Results:**

The results showed that individuals carrying the A allele in the *MAPT* rs1467967 polymorphism, the GG genotype in the *MAPT* rs7521 polymorphism, and the G allele in the *MAPT* rs242557 polymorphism had worse performance on various neuropsychological tests. Carriers of the C allele in *MAPT* rs2471738 polymorphism and CC homozygotes also showed worse performance on neuropsychological tests and pathological levels of several cerebrospinal fluid (CSF) biomarkers. However, T allele carriers in the *MAPT* rs2471738 polymorphism were more represented among patients with dementia and apolipoprotein E (*APOE*) ɛ4 carriers. Carriers of the H2 *MAPT* haplotype had worse performance on various neuropsychological tests, consistent with our previous study, which associated the H2 *MAPT* haplotype with pathological levels of CSF AD biomarkers. Regarding the *MAPT* rs3785883 polymorphism, further research is needed since both the AA and GG genotypes were associated with pathological levels of CSF and plasma AD biomarkers.

**Discussion:**

In conclusion, further genetic studies are needed to elucidate the role of *MAPT* haplotypes and *MAPT* haplotype-tagging polymorphisms in the development of AD.

## 1 Introduction

Alzheimer’s disease (AD) is a complex condition influenced by both genetic and environmental factors. Analysis of tau-positron emission tomography (tau-PET) scans from 1,612 individuals identified four different AD subtypes based on the spread of tau pathology: (1) limbic (apolipoprotein E ɛ4+ [*APOE* ɛ4+], less tau, amnestic, older onset, 32.7% of cases), (2) lateral temporal (more tau, rapidly progressive, multi-domain impairment, asymmetric, 18.1% of cases), (3) medial temporal lobe-sparing (*APOE* ɛ4-, younger onset, dysexecutive, 30.2% of cases), and (4) posterior (older onset, slowly-progressing, visuospatial impairment, 19% of cases, also called posterior cortical atrophy or atypical AD) (Vogel et al., 2021). These findings indicate that additional studies are necessary to elucidate AD’s complex interplay between genetic and environmental factors. According to AlzGene (Bertram et al., 2007), 1,395 studies identified 695 genes associated with AD. In the sporadic form of AD, which accounts for about 99% of disease cases, the main genetic factor that increases the relative risk of developing AD is the ɛ4 variant of the *APOE* gene. One ɛ4 allele increases the risk of developing AD by about three times, while both ɛ4 alleles increase the relative risk by eight to twelve times (Alzheimer's Association, 2020). Numerous other genes associated with sporadic AD have been discovered, but their influence on the risk of the disease is much smaller than that of the *APOE* gene. These genes include *BIN1*, *CLU*, *ABCA7*, *CR1*, *PICALM*, *MS4A6A*, *CD33*, *MS4A4E*, *CD2AP* (Bertram et al., 2007), *TREM2* (Jonsson et al., 2013), and *PLD3* (Cruchaga et al., 2014), among others. These genes were mostly identified in genome-wide association studies (GWAS) that compared many single nucleotide polymorphisms (SNPs) between AD patients and healthy controls (HC).

The microtubule-associated protein tau (*MAPT*) gene, located on chromosome 17q21.3, encodes the tau protein, which, in addition to amyloid β (Aβ), plays a major role in AD. Although AD is considered a secondary tauopathy (i.e., it is not caused by the mutations in the *MAPT* gene, as is the case in primary tauopathies), certain *MAPT* variants have been shown to increase the risk for AD (Myers et al., 2005; Laws et al., 2007; Chang et al., 2014; Yuan et al., 2017; Zhou and Wang, 2017). Moreover, two *MAPT* haplotypes (H1 and H2), which can be defined by six haplotype-tagging SNPs (rs1467967, rs242557, rs3785883, rs2471738, del–In9, rs7521) (Pittman et al., 2005), have been associated with various neurodegenerative disorders, including AD (Myers et al., 2005; Laws et al., 2007; Kauwe et al., 2008; Di Maria et al., 2010; Pastor et al., 2016; Sánchez-Juan et al., 2019). The *MAPT* H1 haplotype has been shown to increase risk, while *MAPT* H2 haplotype reduces the risk for several neurodegenerative disorders (Strickland et al., 2020). In our previous preliminary study, we observed that the levels of cerebrospinal fluid (CSF) biomarkers differed between patients with different genotypes in *MAPT* SNPs (specifically, rs1467967 and rs2471738) and different *MAPT* haplotypes (Babić Leko et al., 2018). This study aimed to further validate the association of *MAPT* haplotype-tagging polymorphisms with AD. We tested whether there is a difference in the results of neuropsychological tests, levels of CSF and plasma biomarkers, and the frequency of *APOE* genotypes among patients with different genotypes in *MAPT* haplotype-tagging polymorphisms. We also examined whether there is a difference in the distribution of *MAPT* haplotype-tagging polymorphisms and *MAPT* haplotypes between patients with AD and HC. Moreover, we analyzed additional AD biomarkers, such as chitinase 3-like protein 1 (YKL-40), S100 calcium-binding protein B (S100B), neurofilament light chain (NfL), and p-tau_181_/Aβ_1-42_ ratio.

## 2 Materials and methods

### 2.1 Subjects

This study included 964 subjects, of whom 220 were recruited at the University Hospital Centre Zagreb. These 220 participants suffered from various types of primary dementia causes: AD, mild cognitive impairment (MCI), frontotemporal dementia (FTD), vascular cognitive impairment/vascular dementia (VaD), mixed dementia (AD+VaD), non-specific dementia (ND), corticobasal syndrome (CBS), and Parkinson’s disease (PD). AD was diagnosed using the criteria of the National Institutes on Aging - Alzheimer’s Association (NIA-AA) (McKhann et al., 2011). The following criteria were used for the diagnosis of other dementia types: for MCI, the criteria of Petersen et al. (1999) and Albert et al. (2011); for FTD, the criteria of Neary et al. (1998); and for VaD, the Hachinski Ischemic Score (Hachinski et al., 1975) and the criteria of the National Institute for Neurological Disorders and Stroke – Association Internationale pour la Recherche et l’Enseignement en Neurosciences (NINCDS-AIREN) (Román et al., 1993).

All patients included in this study provided informed consent for lumbar puncture and participation in the study. Patients underwent neurological examinations with complete blood tests, including levels of thyroid hormones, vitamin B12 and B9, and serology for syphilis and Lyme disease. The Ethical Committee of the Clinical Hospital Centre Zagreb (case no. 02/21 AG, class 8.1–18/82–2 from April 24, 2018) and the Central Ethical Committee of the University of Zagreb Medical School (case no. 380–59–10,106-18-111/126, class 641–01/18–02/01 from June 20, 2018) approved all procedures, which were conducted in accordance with the Helsinki Declaration (World Medical Association, 2013).

A larger cohort of 744 HC older than 65 years of age included participants from the “10,001 Dalmatians project,” part of the Croatian Biobank program (Rudan et al., 2009). Participants provided informed consent for participation in the study, and the Ethical Board of the University of Split, School of Medicine (case no. 2181-198-03-04/10-11-0008 and 2181-198-03-04-19-0022) approved all procedures.

Information on demographic data, determined biomarkers, and the number of *APOE* and *MAPT* genotypes and *MAPT* haplotypes in all included cohorts is presented in Table 1.

**Table 1:** Information on demographic data, determined biomarkers, and the number of *APOE* and *MAPT* genotypes in different cohorts.

		AD	MCI	VaD	FTD	DLB	AD/VaD	PD	CBS	ND	All patients with dementia^#,^*	HC**	HWE *p*-value^$^
Determined biomarkers	CSF	+	+	+	+	+	+	+	+	+	+	−	
	Plasma	+	+	+	+	+	+	+	+	+	+	−	
	Neuropsychological	+	+	+	+	+	+	+	+	+	+	−	
	Genetic	+	+	+	+	+	+	+	+	+	+	+	
*N*		113	53	14	23	8	2	2	1	4	220	744	
Age	Median (25–75th Percentile)	73 (67–77)	70 (59–75)	71 (63–78)	61 (57–65)	71 (68–75)	75	68	51	67 (52–68)	71 (62–76)	71 (67–76)	
Sex	F/M	60/53	27/26	6/8	11/12	2/6	0/2	0/2	1/0	3/1	110/110	433/311	
MMSE	Mean ± SD	20 ± 4.5	25.1 ± 3	22.2 ± 5	16.5 ± 5.2	19 ± 3.7	20.5 ± 5.0	15	27	19.3 ± 5.3	21.1 ± 5	−	
*APOE*	ɛ2ɛ2									1	1 (0.5%)	3 (0.4%)	
	ɛ3ɛ2	9	1	2	2	1		1	1		17 (7.7%)	71 (9.5%)	
	ɛ3ɛ3	58	36	7	15	4	2	1		2	125 (56.8%)	580 (78%)	
	ɛ4ɛ3	36	14	4	5	2	1			1	63 (28.6%)	78 (10.5%)	
	ɛ4ɛ4	7	2	1	1						11 (5%)	5 (0.7%)	
	ɛ4ɛ2	5									5 (2.3%)	7 (0.9%)	
*MAPT* rs1467967	AA	49	23	6	11	3	1	2	1	4	100 (45.5%)		0.990
	AG	53	21	6	11	4	1				96 (43.6%)		
	GG	11	9	2	1	1					24 (10.9%)		
*MAPT* rs242557	AA	16	10		5	1				1	33 (15%)		0.788
	AG	48	24	8	11	4		1		2	98 (44.5%)		
	GG	49	19	6	7	3	2	1	1	1	89 (40.5%)		
*MAPT* rs3785883	AA	10	4	3	1		1				19 (8.6%)	29 (3.9%)	0.768
	AG	34	11	3	10	4		1		1	64 (29.1%)	256 (34.4%)	
	GG	69	38	8	12	4	1	1	1	3	137 (62.3%)	459 (61.7%)	
*MAPT* rs2471738	TT	7	2								9 (4.1%)	21 (2.8%)	0.768
	TC	37	21	6	7	2		2	1	2	78 (35.5%)	184 (24.7%)	
	CC	69	30	8	16	6	2			2	133 (60.5%)	539 (72.4%)	
*MAPT* rs7521	AA	18	13	2	4	3				1	41 (18.6%)	191 (25.7%)	0.876
	AG	65	30	7	17	4	2	1	1	1	128 (58.2%)	362 (48.7%)	
	GG	30	10	5	2	1		1		2	51 (23.2%)	191 (25.7%)	
*MAPT* haplotypes	H1H1	75	40	8	20	8		1	1	3	156 (70.9%)	491 (66%)	0.281
	H1H2	36	12	6	3		2	1		1	61 (27.7%)	233 (31.3%)	
	H2H2	2	1								3 (1.4%)	20 (2.7%)	

^#^This group consists of patients with AD, MCI, VaD, FTD, AD/VaD, DLB, ND, PD, and CBS. ^*^Number of participants (percentage of patients with dementia carrying that genotype relative to the total number of patients with dementia). **Number of participants (percentage of healthy controls carrying that genotype relative to the total number of healthy controls). ^$^Testing for Hardy–Weinberg Equilibrium (HWE) was conducted using the Chi-Square test in SPSS. AD, Alzheimer’s disease; AD/VaD, mixed dementia; *APOE*, apolipoprotein E; MAPT, Microtubule associated protein tau; CBS, corticobasal syndrome; CSF, cerebrospinal fluid; DLB, dementia with Lewy bodies; F, female; FTD, frontotemporal dementia; HC, healthy controls; HWE, Hardy–Weinberg equilibrium; M, male; MCI, mild cognitive impairment; MMSE, Mini-Mental State Examination; ND, nonspecific dementia; PD, Parkinson’s disease; SD, standard deviation; VaD, vascular dementia.

### 2.2 Analysis of cerebrospinal fluid and plasma biomarkers

This part of the analysis was done in 220 participants recruited at the University Hospital Centre Zagreb. All biomarkers were analyzed in CSF, while some were also analyzed in plasma. CSF was collected by lumbar puncture between intervertebral spaces L3/L4 or L4/L5, centrifuged at 2,000 g for 10 min, and stored at −80°C in polypropylene tubes. Thrombocyte-free plasma was extracted from venous blood through a series of centrifugations (first at 1,100 g for 3 min, followed by centrifugation at 5,087 g for 15 min) and stored at −20°C.

The following enzyme-linked immunosorbent assays (ELISA) were used for the determination of biomarkers: tau phosphorylated at threonine 181 (p-tau_181_) (Innotest Phospho-Tau [181P], Fujirebio), Aβ_1-42_ (Innotest β-amyloid1–42, Fujirebio, Gent, Belgium), NfL (Human NF-L/NEFL ELISA Kit, LifeSpan BioSciences, Seattle, WA, USA), S100B (Human S100B, R&D Systems, Minneapolis, MN, USA), and YKL-40 (Chitinase 3-like 1 Quantikine ELISA Kit, R&D Systems). All biomarkers were measured only in CSF, except for S100B and NfL, which were measured in both CSF and plasma.

### 2.3 Determination of polymorphisms

The salting-out method by Miller et al. (1988) was used for the isolation of DNA from venous blood. In the 220 participants recruited at the University Hospital Centre Zagreb, *MAPT* rs1467967, rs242557, rs3785883, rs2471738, del–In9, rs7521, and *APOE* rs7412 and rs429358 SNPs were determined using TaqMan SNP Genotyping Assays (Applied Biosystems) on an ABI Prism 7,300 Real-Time PCR System apparatus (Applied Biosystems, Foster City, CA). The *MAPT* H1/H2 haplotype is defined by the del-In9 deletion in *MAPT* intron 9.

In 744 participants recruited as part of the “10,001 Dalmatians project,” *MAPT* rs3785883, rs2471738, rs7521, rs1800547, and *APOE* rs7412 and rs429358 were determined using Illumina genotyping platforms (CNV370v1, CNV370-Quadv3, and OmniExpressExome-8v1-2_A, Illumina, San Diego, CA). In this cohort, *MAPT* haplotypes were defined by the *MAPT* rs1800547 SNP. *MAPT* rs1467967 and rs242557 polymorphisms were not determined in this cohort of healthy controls. *APOE* ε2, ε3, and ε4 variants were determined by the following combination of *APOE* SNPs: variant ε2 (rs429358 T allele and rs7412 T allele), variant ε3 (rs429358 T allele and rs7412 C allele), and variant ε4 (rs429358 C allele and rs7412 C allele).

### 2.4 Neuropsychological testing

Participants recruited at the University Hospital Centre Zagreb were tested with various neuropsychological assessments, including the Mini-Mental State Examination (MMSE), the modified MMSE test, the Alzheimer’s Disease Assessment Scale-Cognitive subscale (ADAS-Cog), the Picture Pairs Learning and Recall (PPLR) test, the Word Pairs Learning and Recall (WPLR) test, the Visual Association Test (VAT), the Rey–Osterrieth Complex Figure Test (ROCFT), the Clock Drawing Test (CDT), the California Verbal Learning Test (CVLT), the NeuroPsychiatric Inventory (NPI), the Reverse Naming test, the Stroop test, the Visual Reaction Time (VRT) test, and the Boston Naming Test (BNT).

### 2.5 Statistical analysis

Statistical analysis was performed using SPSS 19.0.1 (SPSS, Chicago, IL, USA) and R statistical software (R Core Team, Vienna, Austria). The level of statistical significance was set at *α* = 0.05.

The frequencies of *APOE* genotypes and different diagnoses among subjects with different *MAPT* genotypes and haplotypes were analyzed using a χ^2^-test (Chi-squared test), with Bonferroni correction applied for pairwise comparisons.

Levels of all fluid biomarkers and the majority of variables collected by neuropsychological testing deviated from a normal distribution (*p* < 0.05), except for ADAS-Cog (*p* = 0.07), BNT (*p* = 0.153), PPLR corr (*p* = 0.193), PPLR incorr (*p* = 0.126), PPLR t corr (*p* = 0.470), and PPLR t incorr (*p* = 0.288). Thus, for comparisons of CSF and plasma biomarkers and scores on neuropsychological tests between groups, we used non-parametric tests. First, all variables were compared between groups of patients with different *MAPT* genotypes using the Kruskal-Wallis test (denoted by the letter “H”), followed by *post-hoc* Dunn’s test, which corrects *p*-values for multiple comparisons. In addition to statistical analyses between all genotypes (for example, AA vs. AG vs. GG), *MAPT* genotypes were also grouped, and additional analyses were conducted (for example, AA vs. G allele carriers and GG vs. A allele carriers). CSF and plasma biomarkers and scores on neuropsychological tests were compared between two groups (for example, AA vs. G allele carriers) using the Mann–Whitney *U* test. All statistical analyses were conducted in the group of all patients with dementia (1), AD and MCI patients (2), and AD patients (3).

Variables were analyzed using linear regression when the influence of possible covariates had to be considered. Since the distribution of the majority of variables (CSF and plasma biomarkers and scores on neuropsychological tests) deviated from a normal distribution, variables were logarithmically transformed before linear regression. CSF and plasma biomarkers and scores on neuropsychological tests were considered as dependent variables, while *MAPT* genotypes and haplotypes (and covariates) were considered as independent variables.

## 3 Results

Before analyses, we tested if the variables were associated with sex, age, and *APOE* genotype (ɛ4+ vs. ɛ4-). Regarding neuropsychological tests, none of the variables were associated with sex, age, or *APOE* genotype. Thus, there was no need to consider sex, age, or *APOE* genotype as covariates, and analyses were not controlled for the influence of these factors. On the other hand, fluid biomarkers showed an association with sex, age, and *APOE* genotype. More precisely, CSF YKL-40 (r_S_ = 0.339, *p* < 0.001), CSF NfL (r_S_ = 0.199, *p* = 0.019), and plasma NfL (r_S_ = 0.413, *p* < 0.001) were positively correlated with the age of participants. Moreover, NfL plasma levels were significantly increased in males compared to females (*U* = 2076, *Z* = −2.392, *p* = 0.017), while the p-tau_181_/Aβ_1-42_ ratio was significantly increased in carriers of the ɛ4 *APOE* genotype (*U* = 3,875, *Z* = −4.056, *p* < 0.001).

Additionally, we tested if the interaction between each *MAPT* polymorphism and *APOE* genotype was significantly associated with the tested variables. It was shown that the interaction between rs242557 and *APOE* significantly predicted scores on ADAS-Cog (*β* = 0.163, SE = 0.077, *p* = 0.037). The interaction between rs1467967 and *APOE* predicted scores on ROCFTD (*β* = −0.315, SE = 0.14, *p* = 0.028). The interaction between rs1467967 and *APOE* (*β* = 0.201, SE = 0.081, *p* = 0.015) and rs242557 and *APOE* (*β* = 0.211, SE = 0.081, *p* = 0.010) significantly predicted VAT time. Additionally, the interaction between rs2471738 and *APOE* predicted CSF NfL levels (*β* = 0.168, SE = 0.070, *p* = 0.017). The interaction between *MAPT* haplotype and *APOE* predicted p-tau_181_/Aβ_1-42_ ratio (*β* = −0.419, SE = 0.115, *p* < 0.001). Thus, for these variables, the aforementioned interactions were considered as covariates.

Scores on neuropsychological tests and levels of CSF and plasma biomarkers in patients with different *MAPT* genotypes and haplotypes are presented in Supplementary Tables S1, S2, respectively.

### 3.1 *MAPT* rs1467967 genotype

**Figure 1:**
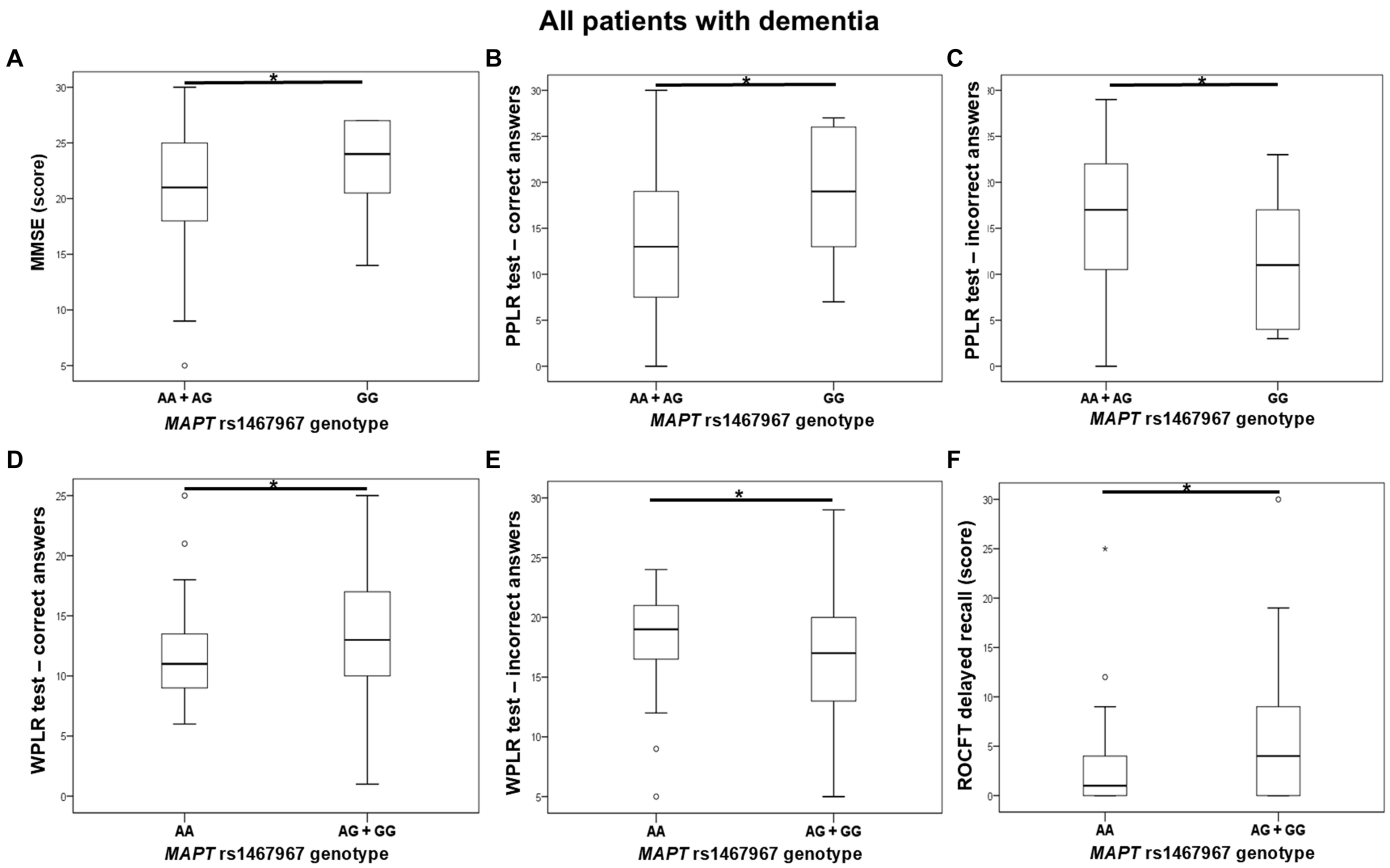
AA homozygotes and A allele carriers in the *MAPT* rs1467967 polymorphism showed poorer performance on **(A)** MMSE, **(B,C)** PPLR test, **(D,E)** WPLR test, and **(F)** ROCFT. The group “all patients with dementia” includes patients with AD, MCI, VaD, FTD, AD/VaD, DLB, ND, PD, and CBS. The box represents the interquartile range (between 25th and 75th percentiles), while the whiskers represent the range between the minimum and maximum values.

Carriers of the A allele in the *MAPT* rs1467967 polymorphism had significantly lower MMSE scores compared to GG homozygotes. Carriers of the A allele had a significantly lower number of correct answers on the PPLR test, and a higher number of incorrect answers compared to GG homozygotes. Moreover, AA homozygotes had a significantly lower number of correct answers on the WPLR test and a higher number of incorrect answers compared to G allele carriers. AA homozygotes also had poorer performance on ROCFT, with lower scores on ROCFT delayed recall compared to G allele carriers (Figure 1; Table 2).

**Table 2:** Association of *MAPT* rs1467967 polymorphism with the results of neuropsychological tests.

Variables	*MAPT* rs1467967	
	A allele carriers vs. GG	G allele carriers vs. AA
MMSE	*U* = 1,662, *Z* = −2.260, *p* = 0.024^A^ (*N* = 217, d_Cohen_ = 0.31, η^2^ = 0.023)	
	*U* = 352, *Z* = −1.960, *p* = 0.050^C^ (*N* = 111, d_Cohen_ = 0.378, η^2^ = 0.034)	
Correct answers on the PPLR test	*U* = 564, *Z* = −2.847, *p* = 0.004^A^ (*N* = 105, d_Cohen_ = 0.408, η^2^ = 0.04)	
	*U* = 387.5, *Z* = −2.050, *p* = 0.040^B^ (*N* = 133, d_Cohen_ = 0.509, η^2^ = 0.061)	
Incorrect answers on the PPLR test	*U* = 582, *Z* = −2.726, *p* = 0.006^A^ (*N* = 133, d_Cohen_ = 0.486, η^2^ = 0.056)	
Correct answers on the WPLR test		*U* = 1852.5, *Z* = −1.958, *p* = 0.050^A^ (*N* = 136, d_Cohen_ = 0.339, η^2^ = 0.028)
		*U* = 1,102, *Z* = −2.098, *p* = 0.036^B^ (*N* = 108, d_Cohen_ = 0.411, η^2^ = 0.04)
Incorrect answers on the WPLR test		*U* = 1841.5, *Z* = −2.006, *p* = 0.045^A^ (*N* = 136, d_Cohen_ = 0.348, η^2^ = 0.029)
		*U* = 1,099, *Z* = −2.117, *p* = 0.034^B^ (*N* = 108, d_Cohen_ = 0.414, η^2^ = 0.041)
ROCFT delayed recall		*U* = 1008.5, *Z* = −2.138, *p* = 0.033^A#^ (*N* = 103, d_Cohen_ = 0.418, η^2^ = 0.042)

Only statistically significant results are presented.^A^all patients with dementia (patients with AD, MCI, VaD, FTD, AD/VaD, DLB, ND, PD, and CBS), ^B^AD and MCI patients, ^C^AD patients.^#^The results remained significant when the analysis was controlled for the influence of the interaction between *MAPT* rs1467967 and *APOE* genotype (β = 0.786, SE = 0.261, *p* = 0.004). In addition to the results of Mann–Whitney *U* test, we provided information on the number of participants (*N*) and effect size (measured by Cohen’s d [d_Cohen_] or eta^2^ [η^2^]). MAPT, Microtubule-associated protein tau; MMSE, Mini-Mental State Examination; PPLR test, Picture Pairs Learning and Recall test; ROCFT, Rey–Osterrieth Complex Figure Test; WPLR test, Word Pairs Learning and Recall test.

Plasma and CSF levels of S100B and NfL, as well as CSF p-tau_181_/Aβ_1-42_ ratio and YKL-40 levels, did not differ significantly between patients with different *MAPT* rs1467967 genotypes.

### 3.2 *MAPT* rs242557 genotype

**Figure 2:**
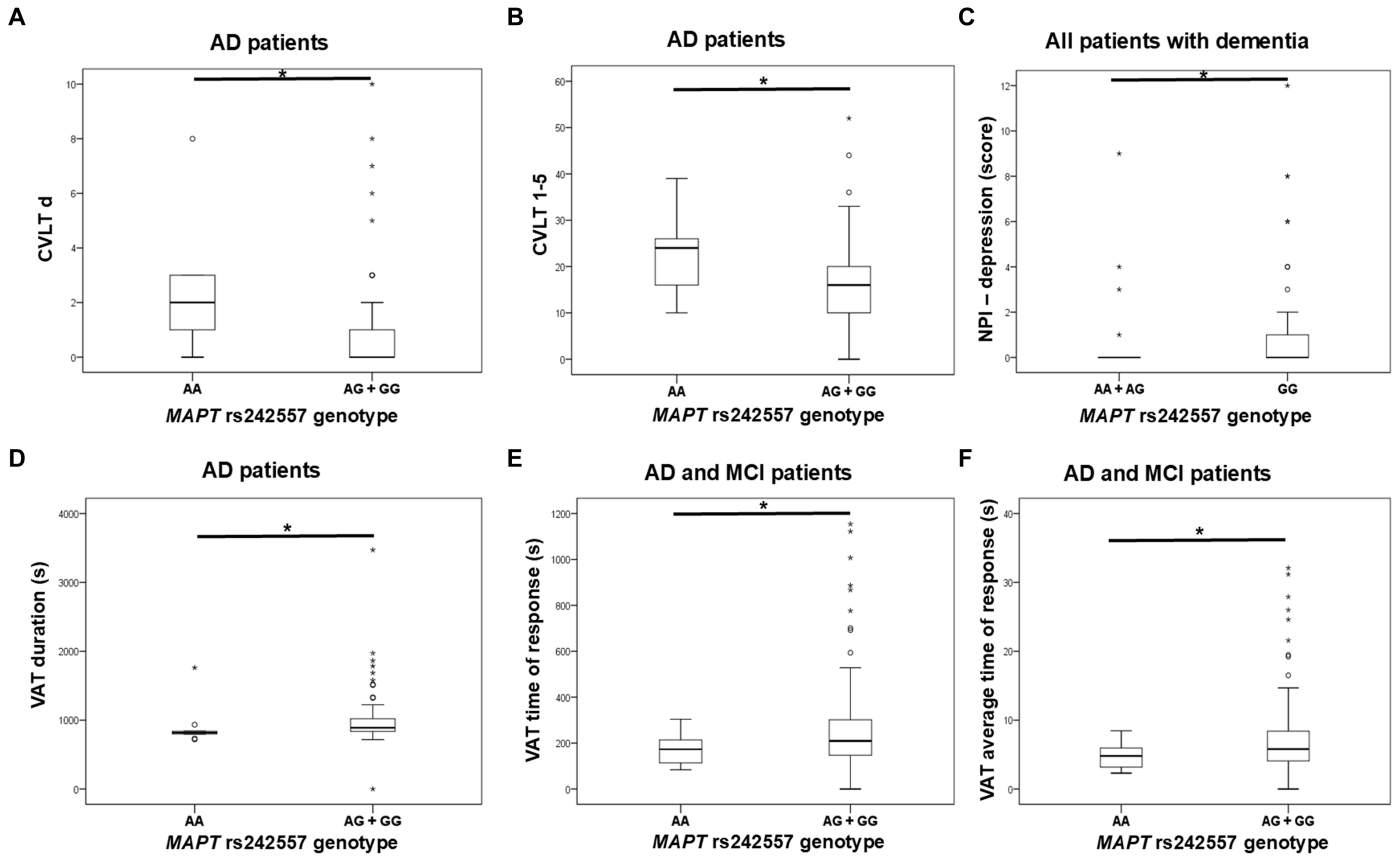
GG homozygotes and G allele carriers in the *MAPT* rs242557 polymorphism showed poorer performance on **(A,B)** CVLT, **(C)** NPI assessment, and **(D–F)** VAT test. The group “all patients with dementia” includes patients with AD, MCI, VaD, FTD, AD/VaD, DLB, ND, PD, and CBS. The box represents the interquartile range (between 25th and 75th percentiles), while the whiskers represent the range between the minimum and maximum values.

G allele carriers in the *MAPT* rs242557 polymorphism had worse performance on the CVLT test compared to AA homozygotes. Specifically, levels of CVLT d and CVLT 1–5 were significantly decreased in G allele carriers compared to AA homozygotes. According to the NPI assessment, GG homozygotes were more depressed than A allele carriers. Moreover, worse performance on VAT (higher levels of VAT, VAT time, and VAT t average) was shown for G allele carriers compared to AA homozygotes (Figure 2; Table 3). PPLR t incorr levels were also higher in carriers of the G allele compared to AA homozygotes.

**Table 3:** Association of *MAPT* rs242557 polymorphism with the results of neuropsychological tests.

Variables	*MAPT* rs242557	
	A allele carriers vs. GG	G allele carriers vs. AA
CVLT d		*U* = 124.5, *Z* = −2.839, *p* = 0.005^C^ (*N* = 65, d_Cohen_ = 0.63, η^2^ = 0.09)
CVLT 1–5		*U* = 147.5, *Z* = −1.987, *p* = 0.047^C^ (*N* = 65, d_Cohen_ = 0.508, η^2^ = 0.061)
NPI - depression	*U* = 950.5, *Z* = −2.793, *p* = 0.005^A^ (*N* = 99, d_Cohen_ = 0.366, η^2^ = 0.032)	
	*U* = 556.5, *Z* = −2.735, *p* = 0.006^B^ (*N* = 78, d_Cohen_ = 0.436, η^2^ = 0.045)	
	*U* = 284.5, *Z* = −2.584, *p* = 0.010^C^ (*N* = 57, d_Cohen_ = 0.52, η^2^ = 0.063)	
VAT		*U* = 138, *Z* = −2.214, *p* = 0.027^C^ (*N* = 66, d_Cohen_ = 0.567, η^2^ = 0.074)
VAT time		*U* = 496, *Z* = −2.027, *p* = 0.043^B#^ (*N* = 108, d_Cohen_ = 0.408, η^2^ = 0.04)
		*U* = 122, *Z* = −2.469, *p* = 0.014^C#^ (*N* = 66, d_Cohen_ = 0.651, η^2^ = 0.096)
VAT t average		*U* = 499.5, *Z* = −2.045, *p* = 0.041^B^ (*N* = 108, d_Cohen_ = 0.401, η^2^ = 0.039)
		*U* = 130.5, *Z* = −2.354, *p* = 0.019^C^ (*N* = 66, d_Cohen_ = 0.606, η^2^ = 0.084)
PPLR t incorr		*U* = 489, *Z* = −1.988, *p* = 0.047^B^ (*N* = 105, d_Cohen_ = 0.396, η^2^ = 0.038)
		*U* = 134, *Z* = −2.141, *p* = 0.032^C^ (*N* = 63, d_Cohen_ = 0.56, η^2^ = 0.073)

Only statistically significant results are presented.^A^all patients with dementia (patients with AD, MCI, VaD, FTD, AD/VaD, DLB, ND, PD, and CBS), ^B^AD and MCI patients, ^C^AD patients.^#^Statistical significance lost after considering interaction between rs242557 polymorphism and *APOE* genotype as covariate. In addition to the results of Mann–Whitney *U* test, we provided information on the number of participants (*N*), and effect size (measured by Cohen’s d [d_Cohen_] or eta^2^ [η^2^]). CVLT, California Verbal Learning Test; MAPT, Microtubule-associated protein tau; NPI, NeuroPsychiatric Inventory; PPLR test, Picture Pairs Learning and Recall test; VAT, Visual Association Test.

No significant difference in plasma and CSF levels of S100B and NfL, as well as CSF p-tau_181_/Aβ_1-42_ ratio and YKL-40 levels, was observed between patients with different *MAPT* rs242557 genotypes.

### 3.3 *MAPT* rs3785883 genotype

**Figure 3:**
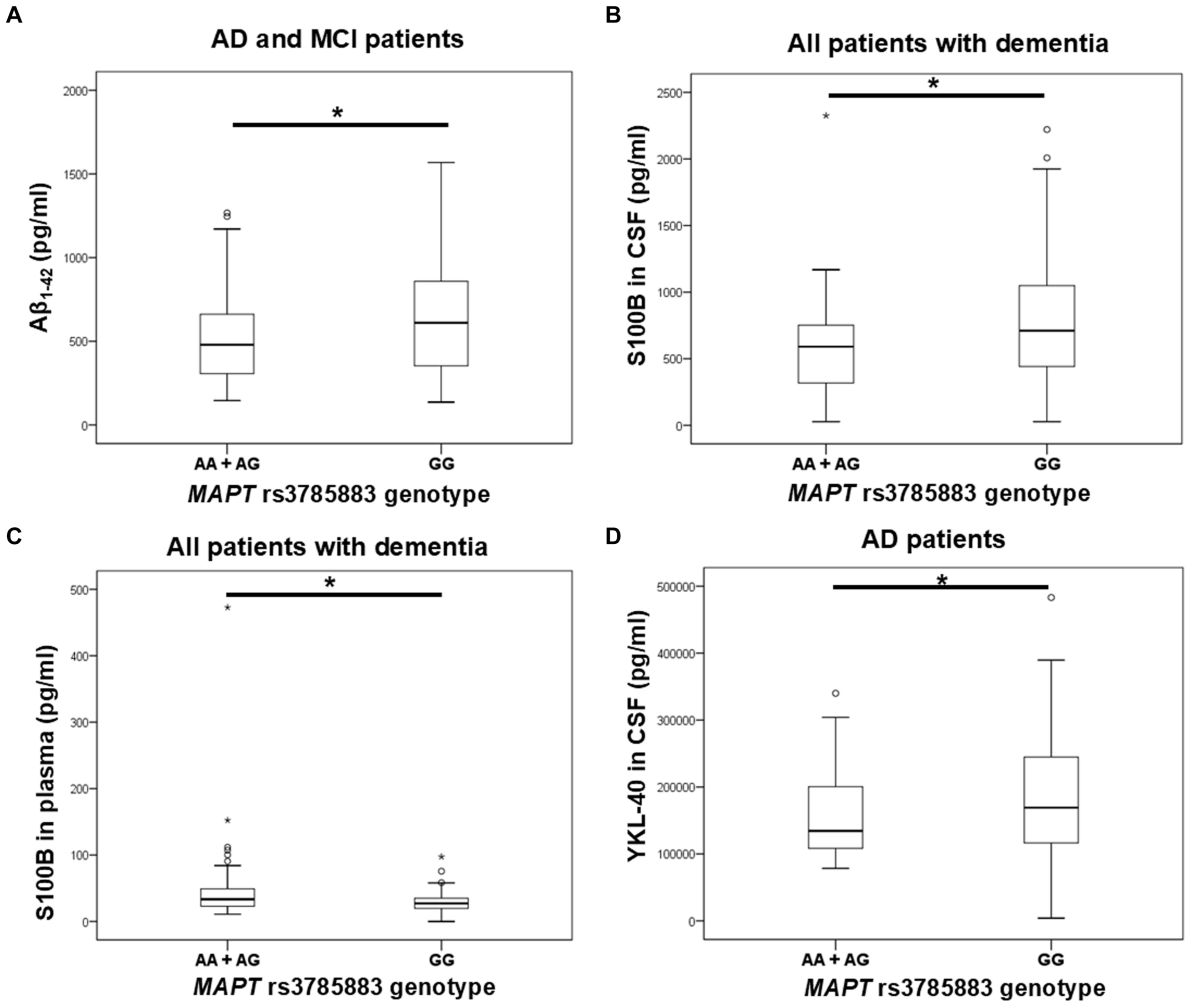
A allele carriers in the *MAPT* rs3785883 polymorphism had significantly decreased Aβ_1-42_ levels **(A)**, and had significantly increased plasma S100B levels **(C)**. Conversely, GG homozygotes in the *MAPT* rs3785883 polymorphism had significantly increased CSF S100B levels **(B)**, and CSF YKL-40 **(D)** levels. The group “all patients with dementia” includes patients with AD, MCI, VaD, FTD, AD/VaD, DLB, ND, PD, and CBS. The box represents the interquartile range (between 25th and 75th percentiles), while the whiskers represent the range between the minimum and maximum values.

Aβ_1-42_ levels were significantly decreased in A allele carriers compared to GG *MAPT* rs3785883 homozygotes (Figure 3; Table 4).

**Table 4:** Association of *MAPT* rs3785883 polymorphism with the levels of fluid biomarkers.

Variables	*MAPT* rs3785883		
	AA vs. AG vs. GG	A allele carriers vs. GG	G allele carriers vs. AA
Aβ_1-42_		*U* = 2,510, *Z* = −2.181, *p* = 0.029^B^ (*N* = 166, d_Cohen_ = 0.344, η^2^ = 0.029)	
Plasma S100B		*U* = 1,541, *Z* = −2.590, *p* = 0.010^A^ (*N* = 135, d_Cohen_ = 0.457, η^2^ = 0.05)	
	*H* = 7.722, df = 2, *p* = 0.021; AG vs. GG (*p* = 0.047)^B^ (*N* = 133, d_Cohen_ = 0.429, η^2^ = 0.044)	*U* = 1,449, *Z* = −2.769, *p* = 0.006^B^ (*N* = 133, d_Cohen_ = 0.495, η^2^ = 0.058)	
CSF S100B	*H* = 7.074, df = 2, *p* = 0.029; AG vs. GG (*p* = 0.033)^B^ (*N* = 135, d_Cohen_ = 0.4, η^2^ = 0.038)	*U* = 1565.5, *Z* = −2.728, *p* = 0.006^A^ (*N* = 137, d_Cohen_ = 0.479, η^2^ = 0.054)	
	*H* = 9.699, df = 2, *p* = 0.008; AG vs. GG (*p* = 0.011)^C^ (*N* = 94, d_Cohen_ = 0.608, η^2^ = 0.085)	*U* = 1531.5, *Z* = −2.636, *p* = 0.008^B^ (*N* = 135, d_Cohen_ = 0.465, η^2^ = 0.051)	
		*U* = 644, *Z* = −3.114, *p* = 0.002^C^ (*N* = 94, d_Cohen_ = 0.678, η^2^ = 0.103)	
YKL-40			*U* = 279.5, *Z* = −2.167, *p* = 0.030^C#^ (*N* = 106, d_Cohen_ = 0.431, η^2^ = 0.044)

Only statistically significant results are presented.^A^all patients with dementia (patients with AD, MCI, VaD, FTD, AD/VaD, DLB, ND, PD, and CBS), ^B^AD and MCI patients, ^C^AD patients.^#^Statistical significance lost after considering the age of participants as covariate. In addition to the results of Mann–Whitney *U* test and Kruskal-Wallis test, we included information on the number of participants (*N*) and effect size (measured by Cohen’s d [d_Cohen_] or eta-squared [η^2^]). Aβ, amyloid β; MAPT, Microtubule-associated protein tau; S100B, S100 calcium-binding protein B; YKL-40, chitinase 3-like protein 1.

There was a significant difference in the distribution of *MAPT* rs3785883 genotypes between HC and patients with dementia (χ^2^ = 9.2, df = 1, *p* = 0.004). Specifically, a higher frequency of AA homozygotes was observed among patients with dementia (*p* = 0.018), while G allele carriers were more represented among HC (*p* = 0.018).

Additionally, A allele carriers had significantly increased S100B plasma levels compared to GG homozygotes (Figure 3; Table 4). Moreover, S100B in plasma was increased in carriers of the AG genotype compared to GG homozygotes (Table 4).

S100B in CSF was significantly increased in carriers of the GG genotype compared to A allele carriers (Figure 3; Table 4). In fact, S100B in CSF was increased in carriers of GG homozygotes compared to carriers of the AG genotype (Table 4). In AD patients, an increase in CSF YKL-40 levels was observed in G allele carriers compared to AA homozygotes (Figure 3; Table 4).

No significant difference in scores on neuropsychological tests was observed between patients with different *MAPT* rs3785883 genotypes.

### 3.4 *MAPT* rs2471738 genotype

**Figure 4:**
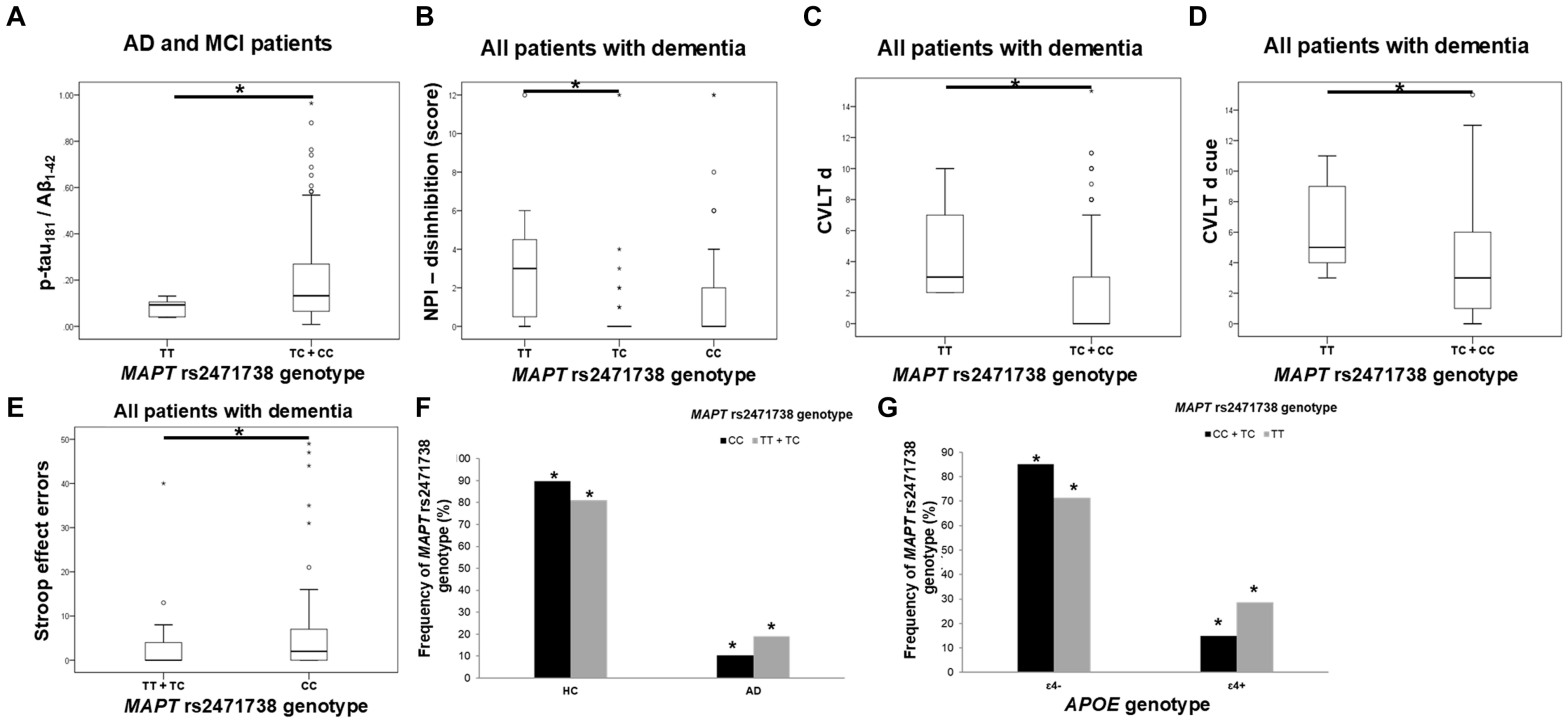
C allele carriers in the *MAPT* rs2471738 genotype had higher levels of p-tau_181_/Aβ_1-42_ ratio **(A)**. TC heterozygotes had a lower rate of disinhibition **(B)** compared to TT homozygotes. C allele carriers and CC homozygotes had worse performance on the CVLT test **(C,D)** and the Stroop test **(E)**. T allele carriers were more frequent among patients with AD **(F)**, and a higher number of *APOE* ɛ4 carriers was observed among TT homozygotes **(G)**. The group “all patients with dementia” includes patients with AD, MCI, VaD, FTD, AD/VaD, DLB, ND, PD, and CBS. The box represents the interquartile range (between 25th and 75th percentiles), while the whiskers represent the range between the minimum and maximum values.

TC heterozygotes in the *MAPT* rs2471738 genotype had a lower rate of disinhibition compared to TT homozygotes. C allele carriers had worse performance on the CVLT test (lower CVLT d and CVLT d cue scores). Finally, CC homozygotes made a higher number of errors on the Stroop test compared to T allele carriers (Figure 4; Table 5).

**Table 5:** Association of *MAPT* rs2471738 polymorphism with the results on neuropsychological tests and the levels of fluid biomarkers.

Variables	*MAPT* rs2471738		
	CC vs. TC vs. TT	C allele carriers vs. TT	T allele carriers vs. CC
NPI - disinhibition	H test = 7.375, df = 2, *p* = 0.025; TT vs. TC (*p* = 0.023)^A^ (*N* = 119, d_Cohen_ = 0.441, η^2^ = 0.046)	*U* = 167, *Z* = −2.139, *p* = 0.032^B^ (*N* = 90, d_Cohen_ = 0.4, η^2^ = 0.038)	
	*H* = 7.765, df = 2, *p* = 0.021; TT vs. TC (*p* = 0.049)^B^ (*N* = 90, d_Cohen_ = 0.533, η^2^ = 0.066)		
CVLT d		*U* = 150.5, *Z* = −2.555, *p* = 0.011^A^ (*N* = 124, d_Cohen_ = 0.598, η^2^ = 0.082)	
		*U* = 489, *Z* = −1.988, *p* = 0.047^B^ (*N* = 95, d_Cohen_ = 0.528, η^2^ = 0.065)	
CVLT d cue		*U* = 174.5, *Z* = −2.106, *p* = 0.035^A^ (*N* = 124, d_Cohen_ = 0.382, η^2^ = 0.035)	
		*U* = 489, *Z* = −1.988, *p* = 0.047^B^ (*N* = 95, d_Cohen_ = 0.456, η^2^ = 0.049)	
Errors on the Stroop test			*U* = 1072.5, *Z* = −2.223, *p* = 0.026^A^ (*N* = 111, d_Cohen_ = 0.414, η^2^ = 0.041)
CSF p-tau_181_/Aβ_1-42_ ratio		*U* = 423, *Z* = −1.962, *p* = 0.050^B#^ (*N* = 163, d_Cohen_ = 0.311, η^2^ = 0.024)	
		*U* = 196, *Z* = −2.061, *p* = 0.039^C#^ (*N* = 112, d_Cohen_ = 0.397, η^2^ = 0.038)	

Only statistically significant results are presented.^A^all patients with dementia (patients with AD, MCI, VaD, FTD, AD/VaD, DLB, ND, PD, and CBS), ^B^AD and MCI patients, ^C^AD patients.^#^Statistical significance lost after considering *APOE* genotype as covariate. In addition to the results of Mann–Whitney *U* test and Kruskal-Wallis test, we included information on the number of participants (*N*) and effect size (measured by Cohen’s d [d_Cohen_] or eta-squared [η^2^]). Aβ, amyloid β; CVLT, California Verbal Learning Test; MAPT, Microtubule-associated protein tau; NPI, NeuroPsychiatric Inventory; p-tau_181_, tau phosphorylated at threonine 181.

The CSF p-tau_181_/Aβ_1-42_ ratio was significantly higher in C allele carriers compared to TT homozygotes (Figure 4; Table 5).

There was a significant difference in the distribution of *MAPT* rs2471738 genotypes between HC and patients with AD (χ2 = 11.889, df = 1, *p* = 0.001), AD and MCI (χ2 = 16.631, df = 1, *p* < 0.001), and patients with dementia (χ2 = 16.226, df = 1, *p* < 0.001). Specifically, a higher frequency of TT homozygotes and T allele carriers was observed among patients with AD (*p* < 0.001, *p* = 0.006, respectively), AD and MCI (*p* < 0.001, *p* = 0.001, respectively), and patients with dementia (*p* < 0.001, *p* = 0.002, respectively). Moreover, among TT homozygotes (χ^2^ = 6.391, df = 1, *p* = 0.024), there was a higher number of *APOE* ɛ4 carriers (*p* = 0.046) (when considering only individuals older than 65 years of age, and after excluding ɛ4ɛ2 genotype from analysis) (Figure 4).

### 3.5 *MAPT* rs7521 genotype

**Figure 5:**
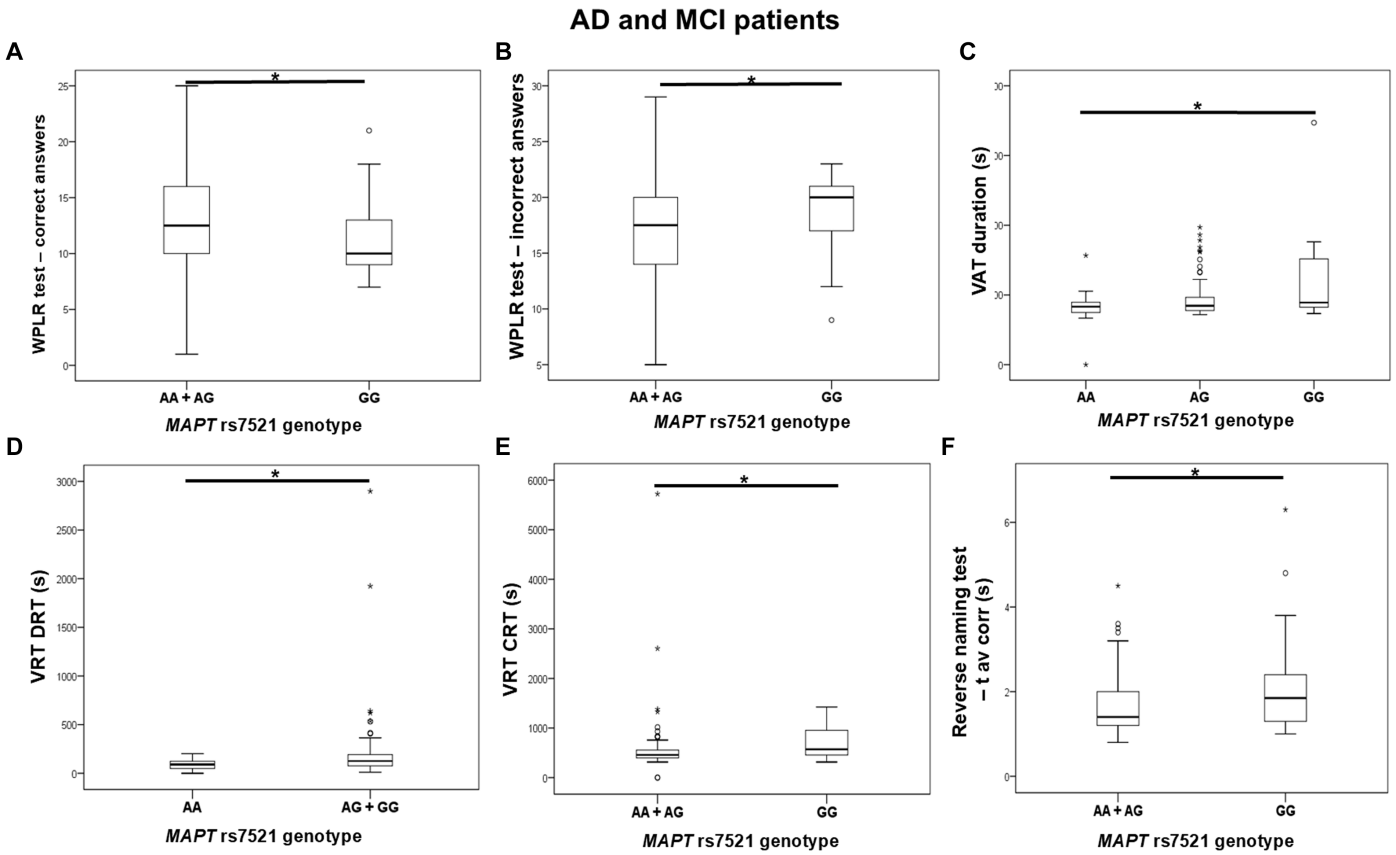
GG homozygotes in the *MAPT* rs7521 genotype had worse performance on the WPLR test **(A,B)**, VAT test **(C)**, VRT test **(D,E)**, and Reverse Naming test **(F)** compared to A allele carriers. The box represents the interquartile range (between 25th and 75th percentiles), while the whiskers represent the range between the minimum and maximum values.

GG homozygotes in the *MAPT* rs7521 genotype exhibited worse performance on the WPLR test. They had a significantly lower number of correct answers on the WPLR test and a higher number of incorrect answers compared to A allele carriers. Furthermore, GG homozygotes had a longer VAT duration on the numbers test compared to AA homozygotes. G allele carriers and GG homozygotes showed poorer performance on the VRT test. Specifically, VRT discrimination reaction time (DRT) was longer in G allele carriers compared to AA homozygotes. The VRT choice reaction time (CRT) was longer in GG homozygotes compared to A allele carriers. Finally, GG homozygotes had a longer average time for giving correct answers in the Reverse Naming test compared to A allele carriers (RN t avcorr) (Figure 5; Table 6).

**Table 6:** Association of *MAPT* rs7521 polymorphism with the results on neuropsychological tests.

Variables	*MAPT* rs7521		
	AA vs. AG vs. GG	A allele carriers vs. GG	G allele carriers vs. AA
Correct answers on WPLR test		*U* = 708, *Z* = −2.583, *p* = 0.010^B^ (*N* = 108, d_Cohen_ = 0.511, η^2^ = 0.061)	
Incorrect answers on WPLR test		*U* = 706, *Z* = −2.597, *p* = 0.009^B^ (*N* = 108, d_Cohen_ = 0.514, η^2^ = 0.062)	
VAT duration on the numbers test	H test = 7.142, df = 2, *p* = 0.028; AA vs. GG (*p* = 0.025)^B^ (*N* = 108, d_Cohen_ = 0.454, η^2^ = 0.049)	*U* = 744, *Z* = −2.314, *p* = 0.021^B^ (*N* = 108, d_Cohen_ = 0.457, η^2^ = 0.05)	
VRT DRT			*U* = 419, *Z* = −2.489, *p* = 0.013^B^ (*N* = 93, d_Cohen_ = 0.534, η^2^ = 0.067)
VRT CRT		*U* = 476, *Z* = −2.163, *p* = 0.031^B^ (*N* = 93, d_Cohen_ = 0.46, η^2^ = 0.05)	
RN t avcorr		*U* = 757.5, *Z* = −2.081, *p* = 0.037^B^ (*N* = 106, d_Cohen_ = 0.411, η^2^ = 0.041)	

Only statistically significant results are presented.^A^all patients with dementia (patients with AD, MCI, VaD, FTD, AD/VaD, DLB, ND, PD, and CBS), ^B^AD and MCI patients, ^C^AD patients.In addition to the results of Mann–Whitney *U* test and Kruskal-Wallis test, we included information on the number of participants (*N*) and effect size (measured by Cohen’s d [d_Cohen_] or eta-squared [η^2^]).MAPT, Microtubule-associated protein tau; RN t avcorr, average time for giving correct answers in the Reverse Naming test; VAT, Visual Association Test; VRT CRT, Visual Reaction Time test choice reaction time; VRT DRT, Visual Reaction Time test discrimination reaction time; WPLR test, Word Pairs Learning and Recall test.

There was no significant difference in the distribution of *MAPT* rs7521 genotypes between AD patients (and also all patients with dementia) and HC. Plasma and CSF levels of S100B and NfL, as well as CSF p-tau_181_/Aβ_1-42_ ratio and YKL-40 levels, did not differ significantly between patients with different *MAPT* rs7521 genotypes.

### 3.6 *MAPT* haplotypes

**Figure 6:**
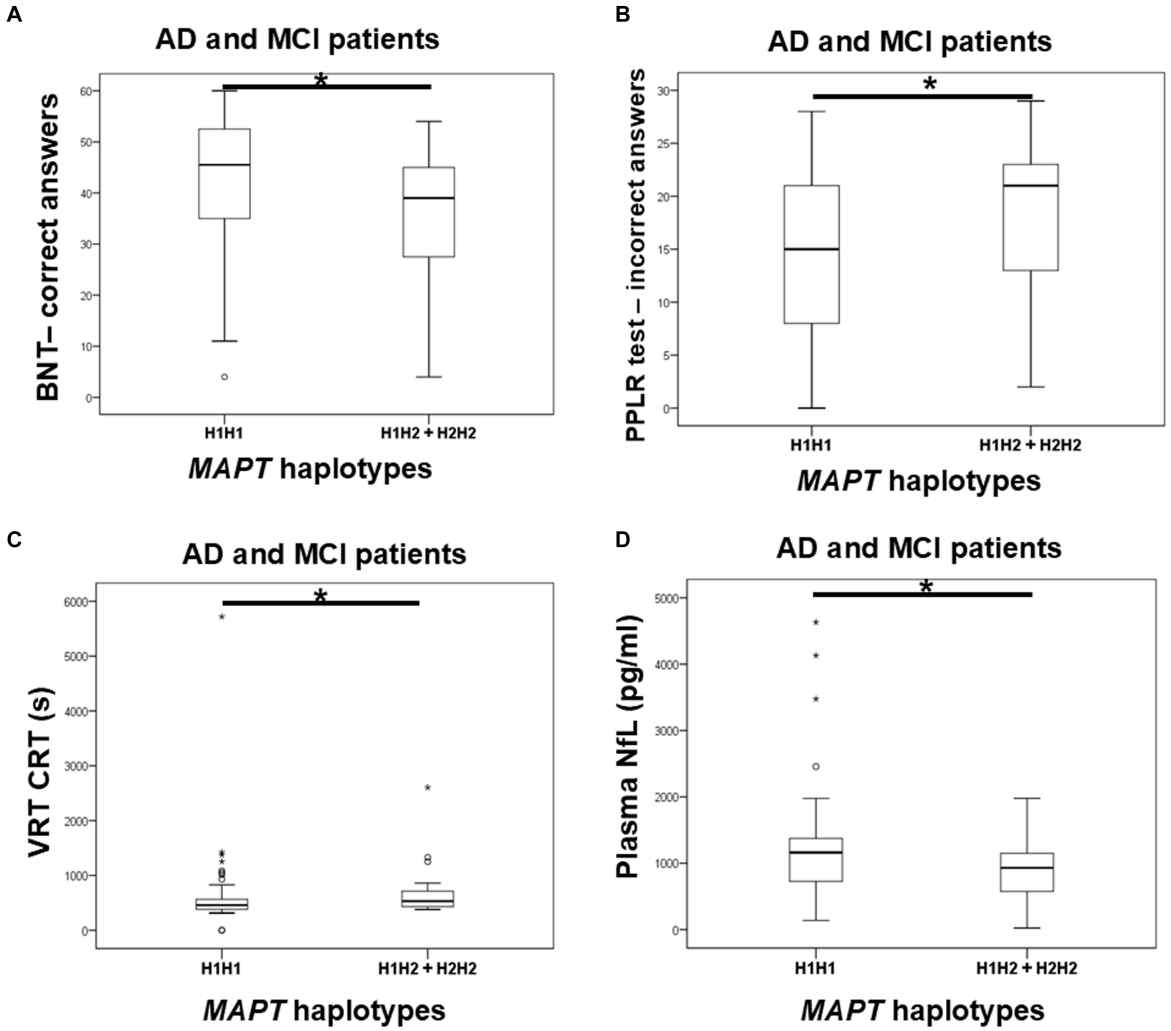
Carriers of the H1H1 *MAPT* haplotype had better performance on the BNT **(A)**, PPLR test **(B)**, and VRT test **(C)**. However, carriers of the H1H1 *MAPT* haplotype had higher plasma NfL levels **(D)** compared to carriers of the H2 *MAPT* haplotype. The box represents the interquartile range (between 25th and 75th percentiles), while the whiskers represent the range between the minimum and maximum values.

Carriers of the H1H1 *MAPT* haplotype exhibited better performance, specifically a higher number of correct answers on the BNT, compared to carriers of the H2 haplotype (H1H2 + H2H2). H1H1 carriers also had fewer incorrect answers on the PPLR test compared to H2 carriers. Finally, H1H1 carriers performed better on the VRT test, with shorter VRT CRT compared to H2 carriers (Figure 6; Table 7). However, H1H1 carriers had higher plasma NfL levels compared to H2 carriers (Figure 6; Table 7).

**Table 7:** Association of *MAPT* haplotypes with the results on neuropsychological tests and the levels of fluid biomarkers.

Variables	*MAPT* haplotypes
	H1H1 vs. H1H2 + H2H2
BNT	*U* = 587, *Z* = −2.623, *p* = 0.009^B^ (*N* = 92, d_Cohen_ = 0.568, η^2^ = 0.075)
Incorrect answers on the PPLR test	*U* = 818.5, *Z* = −2.034, *p* = 0.042^B^ (*N* = 105, d_Cohen_ = 0.405, η^2^ = 0.039)
VRT CRT	*U* = 634, *Z* = −2.029, *p* = 0.042^B^ (*N* = 93, d_Cohen_ = 0.43, η^2^ = 0.044)
Plasma NfL	*U* = 1557.5, *Z* = −2.412, *p* = 0.016^A#^ (*N* = 139, d_Cohen_ = 0.418, η^2^ = 0.042)
	*U* = 1,531, *Z* = −2.374, *p* = 0.018^B#^ (*N* = 137, d_Cohen_ = 0.414, η^2^ = 0.041)
	*U* = 766, *Z* = −2.498, *p* = 0.012^C^ (*N* = 99, d_Cohen_ = 0.519, η^2^ = 0.063)

Only statistically significant results are presented.^A^all patients with dementia (patients with AD, MCI, VaD, FTD, AD/VaD, DLB, ND, PD, and CBS), ^B^AD and MCI patients, ^C^AD patients.^#^The results remained significant when the analysis was controlled for the influence of sex and age (*β* = −0.115, SE = 0.047, *p* = 0.015)^A^, (*β* = −0.111, SE = 0.047, *p* = 0.019)^B^. In addition to the results of Mann–Whitney *U* test, we provided information on the number of participants (*N*), and effect size (measured by Cohen’s d [d_Cohen_] or eta^2^ [η^2^]). BNT, Boston naming test; MAPT, Microtubule-associated protein tau; NfL, neurofilament light chain; PPLR test, Picture Pairs Learning and Recall test; VRT CRT, Visual Reaction Time test choice reaction time.

There was no significant difference in the distribution of *MAPT* haplotypes between AD patients (and also all patients with dementia) and HC. Plasma and CSF levels of S100B, as well as CSF NfL, p-tau_181_/Aβ_1-42_ ratio, and YKL-40 levels, did not differ significantly between patients with different *MAPT* haplotypes.

## 4 Discussion

In the current study, we tested the association of *MAPT* haplotype-tagging polymorphisms and *MAPT* haplotypes with AD. We investigated whether there is a difference in the levels of CSF biomarkers, the results of neuropsychological tests, and the frequency of *APOE* genotypes among patients with different *MAPT* genotypes and haplotypes. Multiple GWA studies were conducted using various CSF AD biomarkers as quantitative traits.^1^ Additionally, various plasma AD biomarkers were analyzed as quantitative traits in GWAS, including plasma p-tau_181_, NfL, Aβ_1-42_, Aβ_1-40_, and t-tau (Stevenson-Hoare et al., 2023). Scores on neuropsychological tests were also used as quantitative traits in AD-associated GWAS (Homann et al., 2022). Due to genetic variability between populations, it is important to replicate studies in different populations. Since GWAS using the aforementioned quantitative traits had not previously been conducted in the Croatian population, we aimed to perform preliminary screening in this study to determine if some of the CSF and plasma biomarkers, as well as scores on neuropsychological tests, are associated with *MAPT* haplotype-tagging polymorphisms and *MAPT* haplotypes.

In this study, we observed that carriers of the A allele and AA genotype in the *MAPT* rs1467967 polymorphism had worse performance on MMSE test, PPLR test, WPLR test, and ROCFT. These results align with our previous study, which showed significantly increased levels of CSF t-tau in AA homozygotes and increased levels of CSF t-tau and p-tau_181_ in carriers of the AG *MAPT* rs1467967 genotype (Babić Leko et al., 2018). Several studies (Mukherjee et al., 2007; Abraham et al., 2009) and two meta-analyses (Yuan et al., 2017; Zhou and Wang, 2017) failed to show an association of the *MAPT* rs1467967 polymorphism with AD. Similarly, the association of the *MAPT* rs1467967 polymorphism with CSF AD biomarkers was not confirmed in studies of Elias-Sonnenschein et al. (2013) and Kauwe et al. (2008). However, Laws et al. (2007) did show an association between the *MAPT* rs1467967 A allele and AD. Ning et al. also noted that the distribution of rs1467967 genotypes differed between VaD patients and HC, suggesting that the *MAPT* rs1467967 polymorphism could serve as a genetic biomarker for VaD (Ning et al., 2011).

GG homozygotes in the *MAPT* rs242557 polymorphism had worse performance on the CVLT test and exhibited more depression than carriers of the A allele. Additionally, G allele carriers had worse performance on the VAT and PPLR tests. In contrast to our observed results, most previous studies associated the A allele of the *MAPT* rs242557 polymorphism with AD. Compta et al. observed increased CSF t-tau and p-tau_181_ levels in PD patients carrying the A allele in *MAPT* rs242557 polymorphism (Compta et al., 2011), while Laws et al. observed increased CSF t-tau levels in AD patients carrying AA *MAPT* rs242557 genotype (Laws et al., 2007). The A allele in the *MAPT* rs242557 polymorphism has been associated with a higher risk for AD (Myers et al., 2005; Laws et al., 2007; Chang et al., 2014; Yuan et al., 2017; Zhou and Wang, 2017), although some studies failed to observe this association (Mukherjee et al., 2007; Abraham et al., 2009; Feulner et al., 2010; Setó-Salvia et al., 2011; Allen et al., 2014). A recent study involving young healthy adults showed that A allele *MAPT* rs242557 carriers had a thinner cortex in brain regions vulnerable to early tau pathology (Huang et al., 2023). Another study conducted in cognitively healthy elders showed that A allele *MAPT* rs242557 carriers had higher retention of the tau-PET tracer ^18^F-AV1451 in the hippocampus, making them more likely to develop tau pathology (Shen et al., 2019). However, a study by Tang et al. conducted in 3072 individuals from China showed that plasma tau levels were increased in participants carrying the G allele in the *MAPT* rs242557 SNP, and GG homozygotes were 1.47 times more likely to develop cognitive impairment compared to AG heterozygotes (Tang et al., 2021).

Carriers of the A allele in the *MAPT* rs3785883 polymorphism had significantly decreased CSF Aβ_1-42_ levels and increased plasma S100B levels. Additionally, a higher frequency of AA homozygotes was observed among patients with dementia. Conversely, GG homozygotes showed a significant increase in CSF S100B and YKL-40 levels. The *MAPT* rs3785883 A allele was associated with an increased risk for AD (Allen et al., 2014), higher levels of CSF p-tau (Kauwe et al., 2008; Cruchaga et al., 2010), earlier age of disease onset (Kauwe et al., 2008), and disease progression (Cruchaga et al., 2010; Peterson et al., 2014). However, some studies reported the association of the *MAPT* rs3785883 G allele with AD (Myers et al., 2005, 2007) or failed to observe any association between *MAPT* rs3785883 and AD (Mukherjee et al., 2007; Abraham et al., 2009; Setó-Salvia et al., 2011). In this study, we confirmed the association of the *MAPT* rs3785883 A allele with CSF Aβ_1-42_ levels, as observed by Kauwe et al. (2008). However, given the significant increase in CSF S100B and YKL-40 levels in GG homozygotes, further validation of this SNP’s association with AD is necessary using a larger cohort of patients. Despite this, compared to other biomarkers, CSF Aβ_1-42_ is considered a much more sensitive biomarker of AD. In our opinion, the association of the A allele with this biomarker is more closely related to the pathological changes that occur in AD.

TC heterozygotes in the *MAPT* rs2471738 genotype showed less disinhibition than TT homozygotes. C allele carriers performed worse on the CVLT test and had higher CSF p-tau_181_/Aβ_1-42_ ratios, while CC homozygotes made more Stroop test errors. More TT homozygotes and T allele carriers were found in patients with AD, AD and MCI, and dementia, with *APOE* ɛ4 carriers being more common among TT homozygotes. Our current study also found more TT homozygotes and T allele carriers among dementia patients and *APOE* ɛ4 carriers, but C allele carriers and CC homozygotes still showed poorer neuropsychological test performance and pathological CSF biomarker levels.

Although *MAPT* rs7521 polymorphism was not previously linked to AD (Mukherjee et al., 2007; Allen et al., 2014; Chang et al., 2014; Yuan et al., 2017; Zhang et al., 2017), we found that GG homozygotes performed worse on the WPLR test, VAT, VRT, and Reverse naming test.

H2 *MAPT* carriers performed worse on the BNT, PPLR, and VRT tests, supporting our previous study results, which showed significantly increased levels of CSF t-tau and p-tau_231_ among carriers of the H2 haplotype (Babić Leko et al., 2018). However, since carriers of H1H1 *MAPT* haplotype showed higher plasma NfL levels, further validation on larger cohorts is needed. Most studies link the H1 *MAPT* haplotype to increased AD risk (Myers et al., 2005; Laws et al., 2007; Kauwe et al., 2008; Di Maria et al., 2010; Pastor et al., 2016; Sánchez-Juan et al., 2019) or suggest the H2 haplotype is protective against AD (Allen et al., 2014; Zhang et al., 2017), though some found no association (Baker et al., 2000; Russ et al., 2001; Mukherjee et al., 2007; Abraham et al., 2009). Interestingly, a recent study involving 338 brain samples of Pick’s disease, a relatively rare and predominantly sporadic form of FTD classified as a primary tauopathy, linked the H2 *MAPT* haplotype to an increased risk of Pick’s disease (Valentino et al., 2023). Geographical differences in *MAPT* haplotype frequency may also help explain the discrepancies in results. In Europe, the H2 *MAPT* haplotype frequency is the highest in Mediterranean countries (Donnelly et al., 2010). In Croatia, which is part of the Mediterranean Basin, the H2 haplotype frequency is over 30% (around 33%). Thus, although we did not observe a significant difference in the distribution of *MAPT* haplotypes between HC and patients with dementia, carriers of the H2 *MAPT* haplotype showed worse performance on neuropsychological tests and pathological levels of various CSF biomarkers. This suggests that some effects of the H2 *MAPT* haplotype on disease progression might not be observed in other populations due to the smaller frequency of H2 *MAPT* haplotype carriers, which is not the case in the Croatian population.

The main strength of our study is the large number of participants and the inclusion of various biomarkers (such as CSF and plasma biomarkers, neuropsychological test, and *APOE* genotype). The main limitation is the cross-sectional design of the study, which does not allow for longitudinal tracking of participants. Additionally, CSF and plasma biomarkers and neuropsychological tests were determined in only 220 participants out of the total number of 964 participants. CSF biomarkers and neuropsychological tests were also determined only in patients with dementia since our “healthy control” group was recruited within a different project, and they have only genetic data.

In conclusion, our study showed that participants with the A allele in the *MAPT* rs1467967 polymorphism, the G allele in the *MAPT* rs242557 polymorphism, and the GG genotype in the *MAPT* rs7521 polymorphism performed worse on various neuropsychological tests. Although T allele carriers in *MAPT* rs2471738 polymorphism were more represented among patients with dementia and *APOE* ɛ4 carriers, C allele carriers and CC homozygotes still showed worse performance on neuropsychological tests and pathological levels of various CSF biomarkers. Carriers of the H2 *MAPT* haplotype also performed worse on various neuropsychological tests, supporting our previous findings that associated the H2 *MAPT* haplotype with pathological CSF AD biomarkers (Babić Leko et al., 2018). Regarding the *MAPT* rs3785883 polymorphism, further research is needed as both AA genotype and GG genotype showed associations with CSF and plasma AD biomarkers, although AA homozygotes were more represented among patients with dementia. Our study further confirmed the association of *MAPT* haplotype-tagging polymorphisms and *MAPT* haplotypes with AD. Some of our results align with mainstream findings, such as the association of the A allele in *MAPT* rs1467967 and rs3785883 polymorphisms with worse performance on neuropsychological tests and pathological CSF Aβ_1-42_ levels, respectively. However, some results differ from most studies, such as the association of the G allele in *MAPT* rs242557 polymorphism and the H2 *MAPT* haplotype with worse performance on neuropsychological tests. Therefore, further research on the association of *MAPT* haplotype-tagging polymorphisms and *MAPT* haplotypes with AD is necessary.

## Data Availability

The data presented in this study are available on request from the corresponding authors.

## Footnotes

^1^https://www.ebi.ac.uk/gwas/
